# The role of universities in enhancing interprofessional collaboration during COVID-19 in Lebanon: academia as neutral convener in a public-private twinning initiative—a qualitative case study

**DOI:** 10.3389/fpubh.2026.1891653

**Published:** 2026-09-03

**Authors:** Kawthar Jaber Fadlallah, Michèle Kosremelli Asmar, Joumana Stephan Yeretzian

**Affiliations:** Center for Collaborative Research Initiatives in Public Health, Higher Institute of Public Health, Faculty of Medicine, Saint-Joseph University of Beirut, Beirut, Lebanon

**Keywords:** academia, COVID-19, interprofessional collaboration, Lebanon, public–private partnership, qualitative case study, twinning

## Abstract

**Background:**

The Coronavirus Disease 2019 (COVID-19) pandemic underscored the importance of interprofessional collaboration, including partnerships between academic institutions and healthcare organizations. Such collaborations, however, are challenging to initiate and sustain during public health emergencies. This study examines the role and perceived effectiveness of academic involvement within a public–private twinning initiative designed to strengthen COVID-19 response capacities in Lebanon.

**Methods:**

Using a qualitative case study design, we conducted 25 semi-structured interviews between January and February 2022 with faculty members, clinicians, hospital administrators, and representatives of the tripartite initiative, as part of a broader study period that began in November 2021 with ethics approval and participant recruitment. Participants were purposively selected based on their involvement in the capacity-building project. Data was analyzed thematically.

**Results:**

Perceptions of academic involvement varied across participant groups. A majority of interviewees (*N* = 21) described the university as a valuable contributor, highlighting enhanced knowledge sharing, improved communication processes, and strengthened capacity-building efforts. Of these, a subset also noted challenges that limited academic contribution (*N* = 12), particularly the timing of the project during the peak of the pandemic, role ambiguity, and communication bottlenecks, indicating that many participants held both positive and critical views simultaneously rather than representing two distinct or opposing groups. Divergent perspectives reflected participants’ professional roles, exposure to research, and organizational context.

**Conclusion:**

Academic engagement was perceived as beneficial when it supported knowledge transfer, coordination, and structured problem-solving. However, its effectiveness was constrained by contextual factors, especially crisis-driven timing and unclear communication pathways. Strengthening academic partnerships in crisis-prone, resource-constrained systems requires early involvement, clear role definition, and sustained channels for intersectoral collaboration.

## Background

Interprofessional collaboration across sectors is widely recognized as essential for responding to the complex challenges posed by the COVID-19 pandemic, challenges that exceeded the preparedness of health systems worldwide (1, 2). These collaborations typically span ministries of public health, education, labor, housing, infrastructure, and social services, alongside private and non-governmental actors (2). Higher education institutions also played a pivotal role. Academic medical centers, faculties, staff, and students mobilized human and material resources, conducted research, supported service delivery, provided training, and facilitated communication and intelligence-sharing across sectors (3, 4). Such contributions enabled the development of innovative and contextually adapted solutions, including clinical support, laboratory capacity, community engagement, and donation of medical equipment (3, 4).

Including universities as partners in multi-sector collaborations offers structural advantages due to their “prestige and environment of a major institution, including its faculty and physical resources” (5). Globally, research universities serve as reservoirs of innovation, capable of influencing public policy and societal progress by generating evidence, training professionals, and convening diverse stakeholders (5). However, evidence shows that engaging academic institutions in public–private partnerships (PPPs) yields both benefits and challenges (2). On the one hand, academic engagement helps align interests across sectors, strengthens communication channels, and fosters intersectoral government support (2). On the other hand, structural barriers such as mistrust among health actors, strict regulations, and resource limitations may hinder collaboration (2). Scholars argue that building a resilient public health system requires sustained investment in partnerships between academia and public health practice (6). Di Ruggiero et al. (6) recommend enhancing collaboration between educational institutions and public health agencies to improve surge capacity, address health inequities, and prepare a trained workforce equipped with updated competencies and equity-oriented leadership skills.

The data presented in this study were collected in January–February 2022, at a relatively early stage in Lebanon’s compounded crises. While several years have since elapsed, the structural dynamics this study documents—the effects of crisis-driven timing on academic-practice collaboration, the disruptive impact of coordinator turnover on institutional memory, and the role of incentive misalignment between public and private actors—are not specific to COVID-19, but represent recurring features of academia’s engagement in health emergencies more broadly. These dynamics remain directly relevant to current global health governance efforts, including the WHO Pandemic Agreement, adopted by the World Health Assembly in May 2025, and the 2024 amendments to the International Health Regulations, which came into force in September 2025 (7, 8). Both instruments explicitly call for strengthened multisectoral collaboration, sustainable workforce capacity, and equitable, coordinated emergency response—priorities that this study’s findings on academia’s role as a neutral convener and capacity-builder directly inform. Furthermore, Lebanon’s health system continues to operate under many of the same resource constraints and structural fragilities documented during the study period, reinforcing the continued relevance of this case for crisis-prone, low-resource health systems more broadly.

Since the acute phase of the COVID-19 pandemic, continued reflection on academia’s role in public health emergencies has extended and refined these findings. A more recent multi-country analysis of academic institutions’ readiness to conduct knowledge translation found that institutional structures, incentive alignment, and leadership stability remain persistent determinants of academia’s capacity to support evidence-informed decision-making during crises (9). Reflecting on a large-scale academic-government modeling partnership established during the COVID-19 response in California, a 2025 commentary similarly identified misaligned incentives, unresolved data-sharing arrangements, and the challenge of sustaining collaboration beyond the acute crisis phase as persistent barriers, even in a well-resourced, high-income setting (10). At the global level, the World Health Organization has recognized that health emergencies routinely produce losses of institutional knowledge and coordination continuity tied to workforce and leadership turnover, and has called for structured mechanisms to preserve experiential knowledge across successive emergencies (11). Broader post-pandemic reflections on the disproportionate toll of COVID-19 in low and middle-income countries have likewise emphasized the need to rebuild trust, strengthen local institutional capacity, and place local actors, including academic partners, at the center of future preparedness efforts (12). Taken together, this growing body of literature confirms that the structural challenges observed in this study, timing, role definition, incentive alignment, and coordination continuity, are not unique to Lebanon or to the acute phase of COVID-19, but recur across academic-practice collaboration in health emergencies more broadly, a challenge now being addressed directly at the global governance level through the WHO Pandemic Agreement and the amended International Health Regulations (see Discussion).

## Twinning in health systems

Twinning is a collaborative partnership model centered on the reciprocal exchange of knowledge and skills between two entities, such as health organizations, clinical teams, universities, or individual professionals, and may involve on-site training, study visits, co-development of technical tools, and the sharing of information and expertise (13). These partnerships often link a more experienced or better-resourced institution with one facing greater operational or systemic challenges, with the goal of fostering mutual learning and building sustainable capacity (14). Unlike short-term technical assistance, twinning emphasizes continuity, peer-to-peer engagement, and the joint creation of context-appropriate solutions (14). This approach has been widely applied across global health to strengthen hospital quality, enhance clinical competencies, and improve management and governance structures. For instance, hospital-to-hospital twinning has been used to reinforce health systems and advance sustainable development initiatives (15), improve diagnosis, treatment, and care for children with cancer in low and middle-income countries (16), and build academic and clinical capacity in fields such as midwifery (13).

### Study context

Lebanon’s first COVID-19 cases emerged in late February 2020 during an unprecedented national crisis marked by severe economic collapse and a large refugee population (17, 18). Early containment measures, including a nationwide lockdown, initially kept case numbers low—approximately 4,300 by July 2020. However, following the reopening of Beirut’s airport and the catastrophic port explosion in August 2020, infections increased rapidly, with the cumulative number of COVID-19 cases exceeding 26,700 by 17 September 2020 (19).

At the time, 27 hospitals (13 private and 14 public) were designated to manage COVID-19, collectively offering 735 beds and 176 ICU beds. With daily infections between 450–650, occupancy surged rapidly. Public hospitals, which historically operate with limited staff and financial resources, struggled more acutely. Only one public hospital in Beirut had adequately trained staff in COVID-19 ICU care. Severe currency devaluation and critical shortages of supplies further hindered response efforts. Public facilities relied heavily on aid from NGOs to procure essential items such as PPE and diagnostic kits, and staffing shortages worsened after more than 760 healthcare workers—half from public facilities—contracted COVID-19 (20–22).

To reinforce healthcare delivery in public hospitals, the WHO Beirut Office proposed a private-public twinning project between health professionals from the private and public sectors. Consequently, a collaborative project was launched through the MEDiterranean Academy for Learning Health Systems (MEDALS), a tripartite collaborative initiative involving The World Health Organization (WHO), the Ministry of Public Health (MoPH), and the Saint-Joseph University of Beirut (USJ) with the Higher Institute of Public Health (ISSP) USJ acting as the implementing partner. In this paper, “MEDALS” refers both to this formal tripartite governance structure and, in the context of study participants, to the operational coordination team through which the twinning project was implemented day-to-day, staffed jointly by academic (ISSP-USJ) and WHO personnel.

The collaboration aimed to bolster the capacity of healthcare workers in public hospitals to combat COVID-19 in the short term, and to establish a sustainable national health system capable of responding to future crises in the long term.

### Research objective

This study examines the perceived role and effectiveness of academic engagement in strengthening interprofessional collaboration between public and private hospitals during Lebanon’s COVID-19 response, within the context of a WHO-supported public–private twinning initiative. Specifically, it investigates how key stakeholders perceived the value that an academic institution brought to this collaboration, the mechanisms through which this value was realized, and the contextual factors that shaped or constrained academic contribution.

This study is guided by the following primary research question: How did stakeholders across academic, clinical, administrative, and international-partner groups perceive the role and effectiveness of academic involvement in a public–private twinning initiative implemented during a compounding public health and economic crisis?

This primary question is addressed through three sub-questions:

- How did perceptions of academia’s contribution and legitimacy within the twinning project vary across different stakeholder groups, and what accounts for this variation?
- Through what specific mechanism—communication, problem-solving capacity, institutional trust, and the translation of theory into practice—did academic involvement add value to the collaboration?
- What contextual and organizational factors (e.g., project timing, incentive structures, and coordination stability) shaped, enabled, or constrained the depth and effectiveness of academic engagement.

## Methods

### Study design and sample

This study employed a qualitative case study design to explore the perceptions of key stakeholders regarding the role of Saint-Joseph University of Beirut (USJ) in enhancing interprofessional collaboration among healthcare professionals involved in a twinning project between public and private hospitals in Lebanon. The research was conducted between November 2021 and February 2022, following approval from the USJ ethics committee (Reference number: USJ 2021-243).

### Population and sample

All stakeholders directly involved in implementing and participating in the twinning project were eligible and invited to participate, reflecting a total population sampling approach that aimed to maximize inclusion and minimize selection bias. Data was collected from those who agreed to take part and were available during the data collection period; individuals who declined or were unavailable were excluded. The study population included representatives from international partners (World Health Organization), academia (Higher Institute of Public Health, USJ), and healthcare professionals and administrators working in public and private hospitals. The Ministry of Public Health (MoPH), while a founding tripartite partner of the MEDALS initiative and the project’s originating stakeholder—motivated by the goal of reducing ICU mortality through strengthened public-hospital capacity—was not directly involved in project implementation activities and was therefore not included among study participants.

### Setting

The study was conducted within the context of a national twinning project implemented in Lebanon, which aimed to strengthen interprofessional collaboration between public and private healthcare institutions during the COVID-19 pandemic. Interviews were conducted either in person or by telephone, depending on participants’ availability and preferences.

### Data collection

Data were collected through semi-structured interviews lasting approximately 30 min (range: 25–35 min). An interview guide was developed based on a review of the literature and iterative discussions among the research team to identify key thematic areas. These included perceptions of the academic institution’s role in the collaboration, its added value, and the barriers and enablers influencing its involvement. The interview guide was pilot tested prior to data collection; questions were deliberately broad and open-ended, allowing participants to shape their own responses rather than being guided toward specific answers.

The interview guide consisted of short open-ended questions designed to encourage reflection and in-depth discussion. Sample questions included: “What is your perception of the role of ISSP–USJ in the twinning project?,” “How did the involvement of ISSP–USJ add value to the collaboration?,” and “What advantages or challenges arise when an academic institution is involved in such partnerships?”

Interviews were conducted by two interviewers—one male and one female—both holding a Master of Public Health (MPH) degree and recruited specifically for this study; their MPH training provided the research methodology and interviewing skills required for qualitative data collection. Neither interviewer had any prior professional or personal relationship with participants, nor had either been involved in the twinning project or the MEDALS initiative. At the start of each interview, the interviewer introduced themselves, explained the aim of the study, and reviewed participants’ rights and the relevant ethical considerations, including voluntary participation, confidentiality, and the right to withdraw at any time, before proceeding. Only the participant and the interviewer were present during each interview; no other individuals attended.

All interviews were conducted in Arabic and audio-recorded with participants’ informed consent. Interviews were carried out face-to-face or by telephone, according to participants’ availability and preference. No repeat interviews were conducted, and no field notes were taken. The two bilingual research assistants, both native Arabic speakers, transcribed the recordings verbatim and translated the transcripts into English. To ensure translation accuracy, a third bilingual researcher independently reviewed all translated transcripts against the original Arabic versions. Any discrepancies were resolved through discussion and consensus prior to analysis.

Of the 52 eligible stakeholders invited to participate, 25 completed interviews, yielding a response rate of 48% (25/52); the remaining 27 individuals withdrew or were unavailable during the data collection period, largely due to the high clinical workload experienced by healthcare staff during this stage of the pandemic response.

### Data analysis

Data were analyzed using thematic analysis, following Braun and Clarke’s six-step framework. A hybrid coding strategy was applied, combining deductive codes informed by the interview guide’s thematic domains with inductive codes that emerged directly from the data. Coding was conducted manually using Microsoft Excel. The two research assistants who conducted the interviews completed the initial coding of the transcribed data, remaining open to codes emerging directly from participants’ accounts. A third researcher, a member of the study’s author team, subsequently reviewed all codes, revising and refining them and organizing them in relation to the thematic domains identified in the interview guide. The resulting codes and their groupings were then discussed with the remaining members of the author team, and the final thematic analysis was developed collaboratively from this iterative process.

Analytical rigor was further supported through this iterative review process—whereby codes generated by the initial coders were checked, revised, and discussed by the wider research team before themes were finalized—and through the use of thick description, achieved by retaining participants’ professional role, institutional affiliation, and the specific circumstances they described alongside quotations presented in the Results, to preserve the context needed for readers to judge the transferability of findings to other settings.

Given the study’s total-population sampling approach, which aimed to include all eligible stakeholders associated with the twinning project rather than to reach saturation through iterative sampling, data saturation was not used as a stopping criterion. Nonetheless, thematic repetition was observed across several codes in later interviews, suggesting reasonable coverage of key perspectives within the study population. Transcripts were not returned to participants for review.

To ensure confidentiality, numerical codes were assigned to all participants, and identifying information was removed from transcripts. Audio recordings and transcripts were stored on password-protected, encrypted devices accessible only to the research team. All data will be retained for 5 years in accordance with USJ ethics committee guidelines and then permanently deleted.

### Reporting standards

This study is reported in accordance with the Consolidated Criteria for Reporting Qualitative Research (COREQ) guidelines (23).

### Reflexivity and positionality

All three authors are researchers affiliated with ISSP-USJ), the academic institution that also served as the implementing partner of the MEDALS twinning initiative under evaluation in this study. This dual positioning—as both researchers and members of the institution being assessed—constitutes a form of insider research and required deliberate methodological safeguards to limit bias in data collection and interpretation. To mitigate this, data collection was conducted entirely by two bilingual research assistants who were independent of the MEDALS project team and had no prior involvement in the twinning initiative; they were recruited specifically for this study’s data collection and had no reporting relationship to, or stake in, the project being evaluated. The authors themselves did not conduct any interviews, reducing the likelihood that participants—several of whom worked directly with ISSP-USJ staff during the twinning project—would shape their responses to align with perceived institutional expectations. Participants were further assured of confidentiality and anonymity prior to each interview, informed that data would be reported using numerical codes with no identifying information retained, and reminded of their right to withdraw at any time without consequence, all of which were intended to encourage candid reporting of both positive and critical perspectives on the university’s role. During analysis, the authors remained attentive to their institutional position as ISSP-USJ researchers and sought to guard against overly favorable interpretation of findings by giving explicit analytic attention to critical and dissenting accounts (e.g., the more reserved assessments offered by WHO representatives), rather than allowing the predominance of positive appraisals to dominate the thematic narrative.

## Results

In total, we interviewed 25 individuals, including physicians, nurses, and executive persons/hospital administrators working in ICU units in participating public and private hospitals, as well as representatives from MEDALS coordination team comprising both academic and WHO staff (Table 1). To preserve participant confidentiality and prevent potential re-identification given the small size and interconnected nature of the twinning project’s stakeholder network, additional individual-level characteristics (e.g., years of experience, exact job titles) are not reported. The Ministry of Public Health, as the initiating partner of the tripartite MEDALS structure, was not directly involved in implementing the twinning project and was therefore not represented among interviewees.

**Table 1:** Distribution of interviewees across categories in the twinning project (*N* = 25).

Category	Medals	Public hospitals	Private hospitals	Total
Academia	7	—	—	7
WHO	2	—	—	2
Physicians	—	—	5	5
Nurses	—	4	4	8
Executive persons	—	—	3	3
Total	9	4	12	25

“MEDALS” refers to the tripartite initiative’s operational coordination team, comprising academic (ISSP-USJ) and WHO staff. The Ministry of Public Health, while a founding tripartite partner, was not directly involved in implementation and is not represented in this table.

### Perceptions of academia’s role in the twinning project

The overall perception of the role of an academic institution in enhancing the collaboration between public and private hospitals emerged as a topic marked by diverse viewpoints. Most participants, including nurses, physicians, and executives, recognized the university’s critical contribution, describing it as “*much needed*.” Accordingly, these views were supported by the fact that the academic institution served as a ¨… *catalyzer of the collaboration*…¨. For instance, one participant declared that ¨…*having the twinning project within the frame of an academic institution was a very correct choice*…¨. In the same context, another participant found that ¨… *academic experience is fundamental and particularly important to the success of a twinning project*…¨. For this group of participants, the academic institution was considered as ¨… *the maestro of the project*…¨, they explained that ¨…*the academic institution is the leader when it comes to the process and the progress of the partnership*…¨. Beyond its primary goal of enhancing healthcare system preparedness for disaster response, the project offered an unexpected benefit: fostering personal development among participants. The project provided learning and training opportunities, which were valuable in fostering a sense of self-development among team members. This, in turn, acted as a powerful influence that ¨…*helps motivate the team members to get more involved and active*…¨ while participating in such projects, according to an executive person.

Moreover, a private hospital physician expressed a distinct perspective on the university’s involvement in the collaborative project, stating. I *believe that the twinning project can be done without the presence of an academic institution. However, the presence of the university helped us achieve more*. This participant emphasized the university’s contribution as the ¨…*icing on the cake*…¨, implying that, the university’s presence significantly elevated the overall outcomes.

In contrast, a few participants, mainly two WHO representatives, described the participation of academics as *“a weak involvement of the university,”* suggesting that the level of engagement did not meet their expectations. Their remarks indicated that although the university was formally part of the project, its contribution was perceived as limited in depth and visibility. Specifically, participants noted that academics were not fully integrated into decision-making processes, did not take a leading role in structuring or evaluating project activities, and did not provide the anticipated research or analytical outputs. As one participant explained, *“we were not able to take advantage of the presence of an academic institution as much as we would have wanted,”* while another expressed disappointment, stating, *“unfortunately, I cannot see any academic input at all,”* implying that greater academic leadership in evidence generation, evaluation, and methodological guidance was expected but not realized.

This pattern suggests that WHO representatives’ expectations of academia were oriented toward a strategic and evaluative function—one centered on generating evidence, shaping decision-making, and providing methodological oversight of the initiative as a whole—rather than the operational, front-line forms of support (communication facilitation, hands-on problem-solving, practical training) that clinical and nursing staff more readily recognized and valued. The gap between these expectations and what was delivered, rather than reflecting an absence of academic contribution altogether, appears to reflect a mismatch between the *kind* of academic involvement WHO representatives anticipated and the *kind* that was actually enacted within the project.

### Advantages of academic involvement in the twinning project

From fostering effective communication and problem-solving to establishing trust and bridging the gap between theory and practice, academic involvement clearly enhanced collaboration and project outcomes.

#### Academic involvement as a means of effective communication

This subtheme concerns the structural and procedural mechanisms through which academic involvement improved the flow of information between institutions—that is, the concrete coordination tools and channels the university introduced, rather than the relational quality of the interactions themselves.

Before the involvement of the academic institution, participants described several communication gaps within the twinning project. These included irregular information exchange between public and private hospitals, inconsistent reporting formats, and the absence of a neutral body responsible for coordinating updates across teams. Communication was often dependent on informal, *ad hoc* interactions, leading to misunderstandings, duplication of efforts, and delays in decision-making.

Participants, including academics, nurses from public and private hospitals, and physicians from private hospitals, highlighted that the academic institution helped to address these issues by establishing structured and consistent communication mechanisms. The university acted as a “*catalyzer,”* facilitating the flow of information through scheduled meetings, standardized documentation, and clear channels for sharing updates. Academics were perceived as neutral intermediaries who helped maintain professionalism and focus in cross-hospital communication, with one participant noting that their presence contributed to *“…keeping the communication between the two hospitals so professional….”* Through these concrete actions, the university provided a coordinated platform for exchanging insights and expertise among stakeholders and ensured that communication became more transparent, organized, and reliable.

#### Academic expertise as a problem-solving asset

This subtheme focuses on the cognitive and analytical contribution of academic involvement—the technical and methodological expertise academics brought to identifying root causes and designing evidence-informed solutions—distinct from the more hands-on, applied translation of these skills described in Section 1.4.

Participants attributed the university’s problem-solving capacity to a combination of its technical expertise, neutral positioning, and leadership skills. Several participants explained that the academic team brought methodological and analytical expertise that enabled them to identify root causes of challenges and propose structured, evidence-informed solutions. In addition, academics were perceived as neutral actors, external to hospital hierarchies, allowing them to mediate disagreements and facilitate balanced decision-making across institutions. Their ability to coordinate tasks, maintain an organized workflow, and provide clear guidance was also described as a form of practical leadership that supported smoother collaboration.

These combined assets, technical knowledge, neutrality, and leadership, were viewed as central to their effectiveness. As one participant noted, *“…the added value of the presence of an academic institution is their capacity to be good leaders and to solve all problems whenever needed….”* This sentiment, echoed by academics and physicians from private hospitals, underscores the academic institution’s multifaceted strengths in driving problem resolution throughout the project.

#### Reputation as a Foundation for Trust

This subtheme addresses the relational and affective outcome of academic involvement—the trust and confidence generated by the university’s neutral position and institutional reputation—as opposed to the concrete communication mechanisms described in Section 1.1, which trust helped to sustain but is not equivalent to.

Trust was repeatedly highlighted by participants, particularly those from academia and nurses in public hospitals, as a cornerstone for the success of projects of this nature. Their comments indicate that academic involvement strengthened several specific dimensions of trust. First, institutional trust increased due to the university’s reputation as a “*well-known and highly acclaimed academic institute.*” Participants associated this reputation with credibility, reliability, and scientific rigor. Second, epistemic trust in the academic team’s expertise and capacity to produce valid knowledge, was reinforced, especially *“when it comes to research and developing teams that can transfer evidence-based knowledge.”* Third, participants described higher interpersonal trust, noting that academics were perceived as neutral, dependable collaborators whose presence helped create a respectful and professional working environment.

#### Academic intervention as a bridge between theory and practice

This subtheme captures the applied, operational translation of academic expertise into hospital teams’ daily workflows—how the analytical contributions described in Section 1.2 were made concrete and actionable in practice, rather than the underlying expertise itself.

The involvement of the academic institution was perceived as a vital mechanism for linking theoretical knowledge to real-world application. Several participants, particularly nurses from both public and private hospitals, emphasized that academia helped translate conceptual tools and evidence-based frameworks into practical, actionable steps during project implementation. For example, academic team members supported frontline staff in operationalizing assessment tools, such as helping nurses apply structured case-analysis templates or standardized triage and referral protocols in the field. Academics also played a key role in interpreting data trends and transforming them into practice-oriented recommendations, such as identifying recurrent patterns in patient flow or documenting system bottlenecks that required immediate operational adjustments. In another instance, academic staff assisted hospitals in developing or refining reporting formats, ensuring that documentation was both analytically sound and practically usable by overstretched clinical teams.

These contributions allowed hospital staff to move beyond abstract training concepts and apply them directly within their daily workflows. As one participant noted, working alongside academics “made the theory tangible,” because academic partners not only provided methodological guidance but also remained available to troubleshoot practical challenges as they emerged. This active, hands-on engagement was viewed as essential to the project’s success, enabling a dynamic exchange between theoretical expertise and field realities.

### Challenges beyond the role of academia

Several external factors influenced the ability of academic institutions to foster collaboration within the Twinning project. These factors affected the collaboration but were not specific to the role of the academic institution and ranged from project timing and resource allocation to communication challenges and team composition. By examining these factors, we gain valuable insights into the contextual hurdles faced by academia and potential areas for improvement in future collaborative endeavors.

#### Project timing

The timing of the project’s launch was identified as a critical factor that limited the academic institution’s ability to contribute fully to the collaboration. Several participants, including academics, executives, and physicians from private hospitals, described the initiative as occurring at the “*wrong timing,”* noting that it was introduced “*in the middle of the crisis, when everyone, especially physicians and nurses, was preoccupied with a high workload in hospitals*.” This intense clinical pressure meant that healthcare staff had limited availability to engage in academic activities such as data collection, training sessions, or joint planning.

From the academic perspective, the timing constrained their contributions by reducing opportunities for early involvement in project design, limiting access to clinicians for coordinated work, and hindering the implementation of structured methodological or evaluative components. Participants explained that the project began after key operational decisions had already been made, restricting the ability of academics to influence initial planning or introduce evidence-based frameworks at the outset.

Several participants also suggested that “*initiating such a project before or after a pandemic could be more advantageous,*” as it would allow for better preparation, more meaningful engagement, and a more stable organizational environment in which academic support could be fully utilized. In this sense, timing affected not only the feasibility of collaboration but also the depth and impact of academic contributions.

#### Uneven distribution of incentives

Participants, including nurses and team members from various institutions, emphasized the challenges posed by the uneven distribution of incentives. Teams from private hospitals, for instance, benefited from financial support, as noted by a nurse from a private hospital who stated *the provision of financial revenues and incentives to our team during the partnership has acted as a facilitator for project implementation*. In contrast, nurses from public hospitals highlighted the absence of similar incentives as a significant barrier, stating that not being able to provide financial support and incentives to team members throughout the project as …*one of the biggest barriers*. Another participant suggested that…*offering financial incentives could have been a valuable enabler, especially during periods of economic and financial crises in Lebanon*. This could have potentially served as a motivating factor and increased commitment in some cases.

#### Management and recruitment conflicts

Management issues within the academic institution were described by participants, including representatives from WHO, physicians from private hospitals, and academic team members, as significant barriers to effective collaboration. Frequent changes in project coordinators disrupted the continuity and stability needed for smooth inter-organizational work. As one participant explained, “*they changed the MEDALS project coordinator two times… I had to deal with three different coordinators, and two of them were not in the medical field… it was confusing.”*

These coordinator changes concretely disrupted collaboration in several ways:

First, loss of institutional memory: Each new coordinator required time to understand the project’s history, objectives, and ongoing activities, resulting in repeated resets in planning and communication. Second, inconsistent guidance: Different coordinators introduced different approaches, expectations, and communication styles, leading to misalignment across teams. Moreover, delayed decision-making: Frequent transitions slowed down approvals, reporting, and problem-solving processes, creating uncertainty for hospital teams. Finally, reduced credibility and clarity: Stakeholders reported confusion about whom to contact, which instructions to follow, and how responsibilities were distributed.

In addition to coordinator turnover, misrepresentation of roles and recruitment of inexperienced team members further complicated collaboration. Participants reported difficulty obtaining accurate information because some newly recruited coordinators lacked familiarity with the project’s goals and did not possess the clinical background required to interpret and relay technical information. As one participant noted, “*most hospital coordinators lacked the necessary skills to report back to our technical team, resulting in a significant loss of crucial information at the beginning of the project.*”

Late recruitment also weakened team cohesion. As another participant remarked, “*the recruitment of team members and coordinators came a little bit late, and they did not have the clinical insights to understand the true purposes of the project.”* A further comment emphasized that “*all team members were learning throughout the project, lacking the necessary background for effective collaboration.”*

## Discussion

This qualitative study explored how key stakeholders perceived the success of the twinning project, with particular attention to the role of academia within the collaboration. Through in-depth interviews, the analysis generated three overarching themes; mixed views on academic involvement, positive influences of academic participation, and challenges and considerations shaping academic contributions, each comprising several subthemes that reflect the complexity of participants’ experiences and evaluations. These themes collectively illustrate how stakeholders understood the added value, limitations, and contextual constraints of academic engagement throughout the project.

### Mixed views: academic involvement in the collaboration

Stakeholders’ divergent views on academia’s contribution are consistent with international literature showing that academic–practice collaborations are rarely experienced uniformly and are shaped by professional identity, power structures, and the degree of exposure to research processes (7, 8, 24). In our study, individuals familiar with research tended to perceive academia more positively, echoing Jessani et al. (8) observation that research literacy facilitates smoother engagement between academic and decision-making actors. Conversely, participants with limited experience in evidence-informed work viewed academia as less visible or insufficiently aligned with operational needs, an issue similarly reported by policy officials in Van der Arend (24) study on research, policy gaps. This variation in perceptions also reflects broader institutional hierarchies. For instance, physicians’ skepticism toward academic involvement and nurses’ greater receptivity mirror patterns documented in the literature on interprofessional collaboration, where established hierarchies shape attitudes toward external partners. These findings suggest that professional role and organizational position significantly mediate the perceived legitimacy of academic collaborators. The particularly critical assessment offered by WHO representatives is also plausibly explained by their distinct structural position within the tripartite MEDALS initiative. As an international convening and oversight body, WHO’s engagement with the project was primarily strategic, focused on ensuring the initiative met its broader policy objectives, generated transferable evidence, and was rigorously evaluated. Academia’s contribution, from this vantage point, was likely judged against a benchmark of methodological leadership, research output, and formal evaluation, functions more aligned with WHO’s own institutional mandate. Physicians and nurses, by contrast, engaged with the project primarily at the operational, clinical level, where academia’s day-to-day value was more visible in the form of coordination support, communication facilitation, and practical problem-solving. This divergence in structural vantage point, one strategic and evaluative, the other operational and clinical, offers a plausible explanation for why WHO representatives perceived academic involvement as comparatively “weak,” even as clinical stakeholders experienced the same involvement as valuable: the two groups appear to have been evaluating academia against different, role-specific standards of what meaningful “academic contribution” should look like.

Importantly, the crisis conditions under which the twinning was implemented appear to have amplified perceptual differences of academic involvement in the collaboration. While previous studies acknowledge contextual constraints on knowledge translation, few have examined how acute emergency settings intensify tensions between strategic and operational modes of collaboration (25). Our findings therefore extend prior work by highlighting how rapid team formation and overwhelming clinical demands can both heighten appreciation for academic structure and exacerbate frustrations when academic contributions are not immediately actionable.

### Positive influences of academic involvement

Where participants perceived clear benefits, these aligned closely with documented facilitators of successful academic–practice collaborations. Trust in the academic institution’s neutrality and reputation echoes findings from Jessani et al. and Rodriguez-Villamizar et al. (8, 26), who highlight institutional credibility as a key factor in partnership acceptance. The value placed on structured problem-solving and methodological support reflects long-standing evidence that academia contributes critical analytical and organizational skills to collaborative initiatives (7). Participants also noted improvements in communication and coordination across institutions following academic involvement. While such observations reflect general facilitators of knowledge-sharing identified in the literature, our findings suggest that these benefits became especially salient in a fragmented health system operating under crisis. In this way, academia appeared to function not only as a technical resource but also as a relational mediator, a role less emphasized in prior scholarship and therefore a unique contribution of this study.

## Challenges and considerations

Several challenges identified by stakeholders’ parallel barriers documented in other academic–practice collaborations, though the present study’s pandemic context adds important nuance. The most prominent concern, frequent turnover of coordinators, echoes findings from Kalbarczyk et al. (25), who examined knowledge translation activities within academic institutions across six low and middle-income countries: Bangladesh, the Democratic Republic of the Congo, Ethiopia, India, Indonesia, and Nigeria. Their multi-country analysis, based on key-informant interviews with representatives from academic institutions and Ministries of Health, highlighted that limited institutional memory, inconsistent leadership, and fluctuating roles were among the most persistent obstacles to sustained evidence-use. In our study, coordinator turnover similarly disrupted continuity, but these disruptions were intensified by the acute pressures of the COVID-19 crisis, where rapid decision-making and overwhelming workloads heightened the impact of leadership instability. This suggests that low-middle income countries’ systems facing emergency conditions are particularly vulnerable to the weaknesses identified by Kalbarczyk et al. (25).

The timing of academic involvement in the twinning project, entering after major operational decisions had already been made, also aligns with literature showing that late-stage or reactive academic engagement can undermine evidence-informed collaboration. Van der Arend (24), in her study of policy officials’ perspectives on the barriers to effective research-policy linkages, found that policy processes often move forward without early academic input, with academic actors frequently brought in only after key agendas and decisions have already been set, limiting their ability to shape strategic priorities. The parallel with our setting is striking: although the institutional landscapes differ, the structural challenge remains similar—academia is positioned downstream rather than upstream, reducing its strategic influence. Participants in our study similarly emphasized that earlier, pre-crisis collaboration would have been far more valuable, allowing teams to establish rapport, clarify roles, and become familiar with tools and procedures before the system was overwhelmed. However, unlike the relatively stable environment described in Van der Arend’s work, our context involved acute pandemic response, where early exclusion had more pronounced consequences.

At the same time, participants acknowledged that certain forms of acute academic support during the crisis, particularly methodological assistance, help interpreting emerging data, and access to university resources, were indispensable, demonstrating that even when upstream engagement is ideal, downstream involvement retains operational value in resource-constrained, crisis-prone settings. Consequently, our findings highlight the importance of both proactive pre-crisis partnership building and flexible crisis-time academic support.

Incentive inequities, particularly disparities between public and private sector compensation, further complicated collaboration in our study. Rodriguez-Villamizar et al. (26), in their qualitative study of academia-government collaboration during the COVID-19 pandemic across five Colombian cities, similarly identified the absence of formal incentive mechanisms for academic collaborators as a key practical gap, recommending that academic institutions urgently establish structures to incentivize and recognize faculty engagement in government partnerships. Notably, academic participants in their study largely framed their own unpaid, voluntary involvement as an act of institutional altruism rather than as an inequity, in contrast to our findings, where comparable disparities in compensation across public and private sectors were experienced more acutely, as a demotivating barrier rather than a source of professional pride. This contrast suggests that the same underlying condition, uncompensated or under-compensated professional contribution, can be experienced differently depending on the broader socioeconomic and institutional context in which academic-practice collaboration unfolds, and highlights the particular vulnerability of Lebanon’s already fragmented, resource-constrained health system to this kind of misalignment.

Finally, participants’ emphasis on soft skills such as communication, empathy, and conflict resolution is consistent with the relational factors highlighted in both Jessani et al. and Kalbarczyk et al. (8, 25). In those studies, participants stressed that interpersonal competencies often determined the success of academic–practice partnerships more than technical capacity. In our context, the salience of these relational skills was heightened by the emotionally charged environment of the pandemic and by the need to mediate tensions between public and private hospitals. This suggests that collaborative efforts in crisis settings may require an even greater focus on relational leadership than collaborations undertaken in more stable environments.

## Relevance to current global health governance: the WHO pandemic agreement and amended IHR

The findings of this study align closely with core provisions of the WHO Pandemic Agreement, adopted by the World Health Assembly on 20 May 2025, and the amended International Health Regulations, which entered into force in September 2025 (7, 8). The Pandemic Agreement explicitly commits Member States to strengthening sustainable, well-trained health emergency workforces and to establishing durable, equitable multispectral collaboration mechanisms in advance of future health emergencies, rather than assembling them reactively once a crisis is underway (8). This study’s finding that late, crisis-driven academic involvement constrained the depth of academia’s contribution directly reinforces the rationale for the Agreement’s emphasis on pre-established, sustained partnerships between academic, governmental, and health-system actors. Similarly, the amended IHR’s strengthened provisions on national capacity-building and equity in implementation resonate with this study’s findings on the disruptive effects of unclear role definition and coordinator turnover, and on the demotivating impact of unequal incentive structures across public and private-sector participants (7). Taken together, the Lebanese case illustrates, at an implementation level, the practical consequences of the very gaps, reactive engagement, ambiguous roles, and inequitable resourcing, that these 2024–2025 global governance instruments now seek to address, and offers empirically grounded lessons for translating their equity and sustainability-oriented aims into practice within crisis-prone, resource-constrained health systems.

## The way forward

While the findings of this study offer valuable insights, the recommendations that follow should not be viewed as universally applicable to all interprofessional collaborations. Instead, they are particularly relevant to crisis-prone, resource-constrained health systems, such as Lebanon’s, where instability, staffing shortages, and fragmented governance create unique challenges for academic–practice engagement. Building on the four cross-cutting priorities identified in this study—strategic project planning, clear communication, incentivizing participation, and leveraging pre-existing partnerships—the following recommendations are organized by stakeholder, translating these priorities into concrete, actionable steps owned, respectively, by the World Health Organization, national ministries of health, and academic institutions.

### Recommendations for the world health organization (WHO)

- Strategic planning: Support the establishment of standing, pre-crisis academic–practice partnership frameworks in member states, rather than commissioning collaborations only once an emergency is underway, consistent with the Pandemic Agreement’s emphasis on sustained, rather than reactive, capacity-building (8).
- Communication: When convening tripartite or multisectoral initiatives, establish clear governance and communication protocols at the outset, explicitly defining the roles and reporting lines of each partner (government, academia, international body) to avoid the ambiguity documented in this study.
- Incentives: Provide technical or catalytic funding support that helps ensure equitable participation incentives across public and private-sector partners in WHO-supported initiatives, particularly in low-resource settings where public-sector compensation structurally lags behind the private sector.
- Pre-existing partnerships: Maintain a registry or mechanism for identifying and rapidly activating existing academic–health system partnerships in member states during emergencies, reducing reliance on newly assembled, unfamiliar teams.

### Recommendations for national ministries of health

- Strategic planning: Invest in longer-term academic–practice collaboration structures during non-crisis periods (e.g., joint training programs, embedded research units within public hospitals) so that academic partners are already integrated into the health system before emergencies arise.
- Communication: Designate a stable, adequately resourced national coordination office for multisectoral health emergency initiatives, with clearly defined decision-making authority, to prevent the disruptive effects of frequent coordinator turnover identified in this study.
- Incentives: Develop harmonized incentive and recognition frameworks for health workers across public and private institutions participating in national capacity-building initiatives, to reduce the demotivating effects of unequal compensation.
- Pre-existing partnerships: Formally recognize and periodically convene existing public–private–academic partnerships outside of crisis periods, so that relational trust and operational familiarity are already established when future emergencies occur.

### Recommendations for academic institutions

- Strategic planning: Proactively seek early, upstream involvement in health system emergency-preparedness planning rather than waiting to be invited once a crisis has begun, and build institutional capacity (e.g., a standing emergency-response unit) to enable rapid, coordinated engagement.
- Communication: Clearly define and communicate the specific scope of the institution’s role, deliverables, and evaluation responsibilities at the outset of any partnership, to manage expectations across stakeholder groups with differing institutional vantage points (e.g., international versus clinical partners).
- Incentives: Advocate internally for non-financial incentive structures, such as professional development opportunities and formal recognition, for faculty and staff engaged in applied crisis-response collaborations, consistent with findings from Jessani et al. (8).
- Pre-existing partnerships: Prioritize the cultivation of longstanding, trust-based relationships with public and private health institutions ahead of any anticipated crisis, drawing on the relational capital emphasized in the broader interprofessional collaboration literature (2).

These stakeholder-specific recommendations address the core challenges identified by participants—coordinator instability, role ambiguity, incentive misalignment, and reactive timing—while offering each type of actor a clear, actionable starting point for strengthening future academic-practice collaborations in crisis-prone, resource-constrained health systems.

## Conclusion

This study offers a nuanced understanding of academic involvement in interprofessional collaboration during a major public health crisis, revealing both its significant added value and its practical limitations. The findings highlight clear benefits—such as improved communication, trust-building, and structured problem-solving—while also underscoring internal tensions related to uneven incentives, coordinator instability, and the timing of project implementation. These mixed perceptions demonstrate that academic engagement is neither uniformly perceived nor uniformly effective; rather, its success is shaped by contextual, organizational, and relational dynamics. Based on these insights, the most crucial and actionable recommendations for strengthening future collaborations include: (1) establishing stable coordination structures that minimize turnover and preserve institutional memory; (2) defining clear roles and communication channels from the outset to reduce ambiguity and streamline decision-making; (3) ensuring equitable incentive frameworks across public and private sectors to support balanced participation; and (4) investing in continuous, pre-crisis academic–practice partnerships so that teams are prepared, trained, and aligned before emergencies arise. These recommendations address the core challenges identified by participants and offer practical steps for enhancing collaboration in similar contexts.

Importantly, this study contributes to pandemic response planning and emergency preparedness by illustrating how academic institutions can serve as neutral conveners, methodological anchors, and communication facilitators during crises. By clarifying the conditions under which academia can meaningfully support the health sector—as well as the barriers that limit its impact—our findings provide a roadmap for designing more resilient, coordinated, and evidence-informed partnerships capable of responding effectively to future public health emergencies.

## Data Availability

The raw data supporting the conclusions of this article will be made available by the authors, without undue reservation.
