# Supplementary material for: The role of universities in enhancing interprofessional collaboration during COVID-19 in Lebanon: academia as neutral convener in a public-private twinning initiative—a qualitative case study
Source: Front Public Health. 2026 Sep 3;14:1891653. doi: 10.3389/fpubh.2026.1891653 (PMC13581759; doi:10.3389/fpubh.2026.1891653)
Supplement: Supplementary file 1 [file Table_1.DOCX]

**Supplementary file 1 – Interview Guide**

| Theme | Questions |
| --- | --- |
| 1. Perceptions of Academia’s Role in the Twinning Project | - How would you describe the role of the academic institution in this twinning project? - What were your expectations regarding academic involvement? - In what ways did the academic institution contribute, or fail to contribute, to the project’s goals? |
| 2. Added Value of Academic Involvement | - In your experience, what were the main benefits or added value of having an academic institution involved in this project? - How did academic involvement influence the way the project functioned or progressed? - Can you describe any positive changes you observed that you attribute to the academic partner? |
| 3. Contextual Challenges Influencing Collaboration | - What major challenges affected collaboration within the twinning project? - In what ways did external factors (e.g., timing, resources, organizational issues) influence the participation of different partners? - Were there any aspects of the project structure or context that made collaboration more difficult? |
| 4. Closing Reflections | - What aspects of academic involvement were most valuable? - What aspects could be improved in future collaborations? - Is there anything else you would like to share about your experience in the project? |
